# Infection of Cerebrospinal Fluid Drainage Devices

**DOI:** 10.1177/10962964251385387

**Published:** 2025-10-06

**Authors:** Kathryn B. Whitlock, Christopher E. Pope, Paul Hodor, David L. Limbrick, Patrick J. McDonald, Jason S. Hauptman, Lucas R. Hoffman, Tamara D. Simon, on behalf of the Cerebrospinal FLuId MicroBiota in Shunts (CLIMB) Study Group

**Affiliations:** 1 New Harmony Statistical Consulting LLC, Clinton, Washington, USA.; 2 Department of Pediatrics, University of Washington, Seattle, Washington, USA.; 3 Aurynia LLC, Seattle, Washington, USA.; 4 Department of Neurosurgery, Washington University in St. Louis, St. Louis, Missouri, USA.; 5 St. Louis Children’s Hospital, St. Louis, Missouri, USA.; 6 Division of Neurosurgery, University of British Columbia, Vancouver, Canada.; 7 British Columbia Children’s Hospital, Vancouver, Canada.; 8 Department of Neurosurgery, University of Washington, Seattle, Washington, USA.; 9 Seattle Children’s Hospital, Seattle, Washington, USA.; 10 Division of Neurosurgery, Barrow Neurological Institute at Phoenix Children’s Hospital, Phoenix, Arizona, USA.; 11 Department of Pediatrics, University of Southern California, Los Angeles, California, USA.; 12 The Saban Research Institute, Los Angeles, California, USA.; 13 Children’s Hospital Los Angeles, Los Angeles, California, USA.

**Keywords:** cerebrospinal fluid, drainage, high throughput sequencing, infection, shunt, ventricular reservoir

## Abstract

**Background:**

Ventricular reservoir infections and cerebrospinal fluid (CSF) shunt infections are diagnosed when bacteria are recovered from microbiological cultures of CSF samples from these devices. We applied high throughput sequencing (HTS) to understand the course of changes in ventricular reservoir and shunt infection microbiota.

**Objectives:**

Evaluate the utility of monitoring microbiota in CSF (1) from ventricular reservoirs to detect development of an infection and (2) during treatment of CSF shunt infections to assess treatment response.

**Methods:**

Study populations included (1) neonates with temporizing ventricular reservoirs who developed reservoir infection and (2) children undergoing treatment for conventional culture-confirmed CSF shunt infection. The V4 region of the 16S ribosomal RNA gene was amplified and sequenced. Comparison of taxonomic results of HTS with standard microbiological culture results (when available) was described for each CSF sample. A robust HTS signal was defined by a microbial load of ≥1e5 microbial genome equivalents/mL.

**Results:**

In none of the five ventricular reservoir infection cases was there a robust HTS signal for the responsible bacteria immediately prior to infection. In six of the seven CSF shunt infection cases, there was a robust HTS signal for the genus of the responsible bacteria in the sample at the time of positive CSF culture. The proportion of sequences from the genus associated with the responsible bacteria decreased during infection treatment.

**Conclusions:**

These pilot data suggest limited utility in using HTS for surveillance for ventricular reservoir infections, as they emerge abruptly. In CSF shunt infection, HTS demonstrates a return to heterogeneous microbiota when bacterial cultures become negative.

## Introduction

Hydrocephalus is one of the most common neurological conditions in the pediatric population. Ventricular reservoirs are devices placed in premature infants to manage hydrocephalus before they are candidates for permanent cerebrospinal fluid (CSF) shunts. CSF is routinely drawn from the ventricular reservoir. The CSF shunt is a more permanent device that has become the mainstay of hydrocephalus treatment for over 60 years.^1^

Both ventricular reservoir infections and CSF shunt infections are diagnosed when bacteria are recovered from microbiological cultures of CSF withdrawn from these devices. Traditionally, bacteria responsible for infection are identified through bacterial culture. The type and the duration of antibiotic treatment are determined by the type of bacteria responsible and duration of positive cultures. Commonly recovered bacteria include the *Staphylococcus* family and other gram-positive bacteria.^2^ To treat infection, the CSF shunt or reservoir is removed, an external ventricular drain is placed for temporary use, a two to three week course of intravenous antibiotic treatment is undertaken, and finally a new CSF shunt or reservoir is placed.^2^

High throughput sequencing (HTS) of 16S ribosomal RNA genes has been adopted recently to identify bacterial species present in the CSF.^3–5^ HTS has revealed the presence of many bacteria and showed us a more complex picture of CSF device infection. Recent studies identified small amounts of DNA from various bacteria and fungi present in the CSF at the time of infection diagnosis, even in the absence of detection by culture.^5^ This data suggest that CSF shunt infection could commonly involve microbes not reliably detected by conventional bacterial cultures, which can only be seen by molecular microbiological tools. With technological advances in these tools and the laboratory and computation pipelines involved, sensitivity and specificity have been significantly increased.^3^ Also, more rigorous exclusion of contaminants has been added in order to reliably identify the microbes responsible.^3^ Considering these innovations, we sought to apply HTS to understand ventricular reservoir and shunt infection. The key to optimizing infection treatment may be to more effectively target the complex microbial milieu present in CSF device infection, hereafter referred to as microbiota.

Therefore, the objectives of this pilot study were to evaluate the utility of monitoring microbiota in CSF (1) from ventricular reservoirs to detect development of an infection and (2) during treatment of CSF shunt infections to assess treatment effectiveness. For ventricular reservoir infections, we hypothesized that (1) increased bioburden and microbial diversity would be associated with longer duration of ventricular reservoir placement and (2) infecting organisms identified by CSF culture would be associated with higher relative abundance of the same genera by HTS. For CSF shunt infection, we hypothesized that (1) infecting organism(s), as identified by traditional culture, would be present at relatively higher abundance at the beginning of infection and (2) overall bacterial load and relative abundance of infecting organism would decrease over the treatment course.

## Design/Methods

### Study population and design

This pilot study capitalized upon hydrocephalus research programs at one of three pediatric centers in the CLIMB study^6^ (St. Louis Children’s Hospital) and an earlier related study at Primary Children’s Hospital.^5^ All CSF samples were collected prospectively.

The first study population included neonates with hydrocephalus being treated by placement of a temporizing ventricular reservoir, who developed potential reservoir infection, defined as identification of organisms on microbiological culture of CSF fluid obtained from a ventricular reservoir during routine treatment; this is throughout referred to as “reservoir infection” given the uncertainty of this clinical diagnosis. Neonates were enrolled at Saint Louis Children’s Hospital from 2009 to 2013 (n = 5).

A second study population included children with hydrocephalus who were under the age of 18 and undergoing treatment for conventional culture-confirmed CSF shunt infection, defined as identification of organisms on microbiological culture of CSF fluid obtained from a partial or complete CSF shunt system. CSF shunt system(s) included ventriculoperitoneal, ventriculoatrial, ventriculopleural, arachnoid cyst shunts, subdural shunts, and lumboperitoneal shunts and excluded temporizing devices. We selected cases with multiple positive CSF cultures to better understand the correlation between traditional bacterial culture and HTS. All of the infection cases were surgically treated with shunt removal and extraventricular drain placement on either day 0 or day 1. Children were enrolled at Primary Children’s Hospital in Salt Lake City, Utah, from 2008 to 2015 (n = 7).

### Ethics statement

All participating centers obtained IRB approval (British Columbia Children’s Hospital, Seattle Children’s Hospital, and St. Louis Children’s Hospital), as well as Children’s Hospital Los Angeles, for participation in CLIMB. This study also received Institutional Review Board approval from the University of Utah, as well as approval from the Primary Children’s Hospital Privacy Board. For all study subjects, except those from Primary Children’s Hospital prior to March 18, 2010, written consent was obtained from parents or guardians, and assent, when age- and developmentally appropriate from study subjects, for additional CSF to be collected on each occasion that regular CSF samples were obtained during treatment for CSF shunt infection. Prior to March 18, 2010, at Primary Children’s Hospital as well as at St. Louis Children’s Hospital, we used CSF remaining after routine processing and testing in the hospital microbiology laboratory.

### Clinical data

Clinical data were abstracted from the medical record for each child at participating centers by trained extractors. Data included demographics, etiology of hydrocephalus, details of surgical treatment, antibiotic treatment, and CSF microbiology lab results.

Cohort characteristics were summarized overall. Categorical variables were described using frequencies and percentages, and continuous variables were described by median and interquartile range (IQR) or mean and standard deviation (SD).

### CSF specimen collection

Sterile conditions were standard practice throughout recovery and storage of CSF for both patient cohorts. For children with the ventricular reservoirs, CSF samples were routinely collected from drainage of ventricular reservoir during standard care for these patients. For children with CSF shunt infection, the first CSF sample for diagnosis of infection was usually obtained from needle aspiration of the shunt reservoir under sterile conditions outside the operating room in a bedside “shunt tap.” The initial CSF sample analyzed in this study either was left over from this first diagnostic sample or was obtained in the operating room under sterile conditions from the system being removed during the first operation to treat infection. Subsequent CSF samples, including those at the end of shunt infection, were generally obtained under sterile bedside conditions through a sampling port within sterile extension tubing attached to the external ventricular drain. CSF samples were stored at 4°C for up to five days. CSF was then aliquoted into vials of ∼100 μL for the study and stored at −70°C.

### CSF sample analysis

Unless otherwise indicated, all CSF samples underwent standard clinical microbiological cultures. All CSF samples were tested using routine aerobic culture techniques in hospital-certified laboratories following Clinical and Laboratory Standards Institute guidelines.

DNA from CSF samples and extraction controls was purified using the AGOWA magMini DNA isolation kit (AGOWA, LGC Genomics, Berlin, Germany) in accordance with the manufacturer’s recommendations and with modifications. Extraction methods were optimized and described in detail previously.^3,7^ CSF sample processing order was randomized. No sample controls were extracted alongside each set of CSF samples. These controls underwent the same procedures as CSF samples and served as DNA extraction kit blanks.

The total bacterial loads for all CSF samples were quantified using broad-range 16S rRNA gene quantitative PCR (qPCR) using appropriate positive and negative controls as previously described.^3,7^

The presence and relative abundance of microbiota in CSF samples was analyzed as described.^3^ The V4 region of the 16S ribosomal RNA gene was amplified and sequenced. After denoising with the DADA2 program, sequences were aligned to the SILVA 16S reference database (v. 132) for taxonomic assignment at the genus or family level. Contaminant sequences were identified and removed using the decontam R package.^8^ An arbitrary cutoff of 10^5^ genome equivalents (GE)/mL was chosen, above which the HTS signal was considered robust. This cutoff represents the 81^st^ percentile of extraction controls (4.29 × 10^4^ ± 6.45 × 10^4^ GE/mL). Comparison of taxonomic results of this analysis with standard microbiological culture results (when available) was described for each CSF sample. Microbiota was characterized within patients and across infection episodes. Sequence incidence was calculated for each sample and summarized across samples.

## Results

We identified five neonates with hydrocephalus being treated by placement of a temporizing ventricular reservoir who developed reservoir infection and had samples available for analysis. The neonates averaged 0.8 months of age at reservoir placement and 2.7 months of age at reservoir infection and had a mean gestational age of 25 weeks and birth weight of 700 grams (Table 1). A range of 4–27 CSF samples were analyzed per reservoir infection. A total of 98 unique V4 sequences were identified after removal of contaminants, representing 43 taxa (genera and families).

**Table 1.** Demographics of Patients in Reservoir Infection and Shunt Infection Groups

Variable	Reservoir infection group	Shunt infection group (*n* = 7)
	(*n* = 5)	
Age at reservoir placement in months, mean [standard deviation (SD)]	0.8 (0.8)	N/A
Age at reservoir infection in months, mean (SD)	2.7 (1.0)	N/A
Age at shunt placement in months, median (IQR)	N/A	5.1 (0.7, 183.1)
Age at initial shunt infection in months, median (IQR)	N/A	5.8 (1.3, 193.5)
Sex, n (%)		
Male	2 (40%)	4 (57%)
Female	3 (50%)	3 (43%)
Race, n (%)		
Black or African American	4 (80%)	0 (0%)
White	0 (0%)	7 (100%)
Unknown or not reported	1 (20%)	0 (0%)
Ethnicity, n (%)		
Latino	0 (0%)	0 (0%)
Not Latino	4 (80%)	7 (100%)
Unknown or not reported	1 (20%)	0 (0%)
Insurance, n (%)		
Public	4 (80%)	4 (57%)
Private	1 (20%)	2 (29%)
Self-pay	0 (0%)	0 (0%)
Unknown	0 (0%)	1 (14%)
Etiology of hydrocephalus, n (%)		
Aqueductal stenosis	3 (60%)	0 (0%)
Myelomeningocele	2 (40%)	1 (14%)
Post-intraventricular hemorrhage	0 (0%)	3 (43%)
Posterior fossa cyst	0 (0%)	1 (14%)
Unknown	0 (0%)	2 (28%)
Gastrostomy, n (%)	0 (0%)	1 (14%)
Tracheostomy, n (%)	0 (0%)	0 (0%)
Complex chronic conditions count, n (%)		
Zero	1 (20%)	4 (57%)
One	4 (80%)	2 (29%)
Two or more	0 (0%)	1 (14%)
Birth weight in kilograms, mean (SD)	0.7 (0.1)	2.1 (1.2)
Gestational age in weeks, mean (SD)	25 (2)	32 (6)

The proportion of bacterial taxa identified by HTS over the course of the five ventricular reservoir infections and bacteria grown in culture on day 0 are shown in Figure 1. The responsible organism from the bacterial culture on day 0 is designated in the header; for example, patient R2’s CSF grew *Streptococcus viridans* on day 0. Relative abundance of HTS results from day −44 to day −1 are provided along the x-axis. Bacterial culture results are compared to HTS findings for the day of infection and the preceding CSF sample in Table 2. Bacterial quantitation by qPCR detected no or minimal bacterial DNA identified above the assay’s limits of detection. In none of the ventricular reservoir infection cases was there a robust HTS signal for the responsible bacteria in the sample immediately prior to infection.

**FIG. 1.**
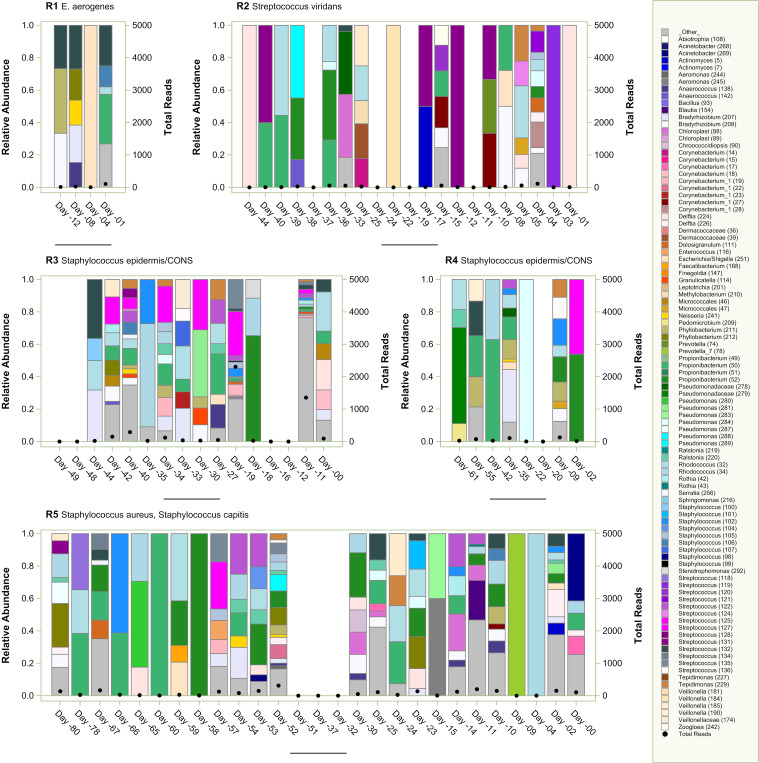
High throughput sequencing results of microbiota for ventricular reservoir infections. Relative abundance is demonstrated with bars with scale on the left-sided y-axis and total reads are shown with heavy black dots and with the scale on the right-sided y-axis. Bacterial culture results on day 0 are provided next to the case identifier (R#).

**Table 2.** Bacterial Culture, High Throughput Sequencing (HTS) Results (Genus/Family, Read Count, % of Reads, and Rank), and qPCR Levels (GE/mL CSF) for Patients with Reservoir Infection and Shunt Infection. Bolded qPCR Indicates GE/mL CSF Was Above 10^5^

Patient	Infection episode day	Infection culture organism	Infection HTS genus/family	Infection HTS *N*	Infection HTS %	Infection HTS rank	qPCR GE/mL CSF
			Reservoir infection				
R1	−4	Unknown	*Escherichia*	5/5	100%	1	1.31 × 10^4^
R1	−4	Unknown	*Enterobacteriaceae*	0/5	0%	2	1.31 × 10^4^
R1	−1	*Enterobacter aerogenes*	*Enterobacteriaceae*	0/108	0%	*	3.07 × 10^4^
R2	−1	Unknown	*Delftia*	5/5	100%	1	1.73 × 10^4^
R2	−1	Unknown	*Streptococcus*	0/5	0%	2	1.73 × 10^4^
R2	0	*Streptococcus viridans*	Unknown	—	—	N/A	N/A
R3	−11	Unknown	Other	1038/1357	76%	1	3.69 × 10^4^
R3	−11	Unknown	*Staphylococcus*	21/1357	2%	9	3.69 × 10^4^
R3	0	*Staphylococcus epidermidis*	*Staphylococcus*	0/91	0%	9	2.13 × 10^4^
R4	−2	Unknown	*Propionibacterium*	7/13	54%	1	2.78 × 10^4^
R4	−2	Unknown	*Staphylococcus*	0/13	0%	3	2.78 × 10^4^
R4	0	*Staphylococcus epidermidis*	Unknown	—	—	N/A	N/A
R5	−2	Unknown	Other	58/154	38%	1	2.72 × 10^4^
R5	−2	Unknown	*Staphylococcus*	6/154	4%	8	2.72 × 10^4^
R5	0	*Staphylococcus capitis, S. aureus*	*Staphylococcus*	44/106	42%	1	3.44 × 10^4^
			Shunt infection				
S1	1	*Staphylococcus aureus*	Unknown	—	—	N/A	N/A
S1	2	*Staphylococcus aureus*	*Staphylococcus*	697/708	98%	1	5.72 × 10^4^
S1	3	*Staphylococcus aureus*	*Staphylococcus*	1436/5797	25%	2	3.76 × 10^4^
S2	1	*Staphylococcus aureus*	*Staphylococcus*	121173/121505	>99%	1	**3.11 × 10^7^**
S2	2	*Staphylococcus aureus*	*Staphylococcus*	5/93	5%	7	<1.54 × 10^4^
S2	6	*Staphylococcus aureus*	*Staphylococcus*	108/610	18%	2	2.68 × 10^4^
S3	1	*Staphylococcus aureus*	*Staphylococcus*	2815/3667	77%	1	**4.66 × 10^5^**
S3	3	*Staphylococcus aureus*	*Staphylococcus*	2144/3456	62%	1	3.73 × 10^4^
S3	5	*Staphylococcus aureus*	*Staphylococcus*	1416/2393	59%	1	**2.04 × 10^5^**
S4	1	*Staphylococcus capitis*	*Staphylococcus*	11895/15155	78%	1	5.54 × 10^4^
S4	2	*Staphylococcus capitis*	*Staphylococcus*	2954/3691	80%	1	2.32 × 10^4^
S5	1	*Staphylococcus epidermidis*	Unknown	—	—	N/A	N/A
S5	2	*Staphylococcus epidermidis*	*Staphylococcus*	89/282	32%	1	<1.54 × 10^4^
S5	3	*Staphylococcus epidermidis*	*Staphylococcus*	834/11172	7%	3	2.24 × 10^4^
S6	1	*Klebsiella pneumoniae*	*Klebsiella*	0/62307	0%	5	**4.97 × 10^6^**
S6	2	*Klebsiella pneumoniae*	*Klebsiella*	0/159078	0%	7	**2.19 × 10^8^**
S6	3	*Klebsiella pneumoniae*	*Klebsiella*	0/38739	0%	3	**7.00 × 10^7^**
S7	1	*Staphylococcus epidermidis*	*Staphylococcus*	10/15	67%	1	<1.54 × 10^4^
S7	2	*Staphylococcus epidermidis*	*Staphylococcus*	541/1903	28%	2	1.65 × 10^4^
S7	3	*Staphylococcus epidermidis*	*Staphylococcus*	9/25	36%	1	2.71 × 10^4^

Significant findings are in bold.

We identified 7 children with hydrocephalus who developed CSF shunt infection and had samples available for analysis. The children had a median age of 7 months of age (IQR 3 to 55 mo) at initial shunt placement (Table 1). A range of 8–16 CSF samples covering days 1–20 post-infection were analyzed. A total of 66 unique V4 sequences were identified after removal of contaminants, representing 48 taxa.

The proportion of bacterial taxa identified by HTS over the course of treatment for the 7 CSF shunt infections and bacteria grown in culture are shown in Figure 2. The responsible organism from bacterial culture and duration of positive culture is designated in the header; for example, S1’s CSF grew *Staphylococcus aureus* on days two through five, was not available on day six, and demonstrated no growth thereafter. HTS was successfully performed on days two through six of infection treatment; these identified a preponderance of *Staphylococcus* sequences both before (top panel) and after decontam R (bottom panel).

**FIG. 2.**
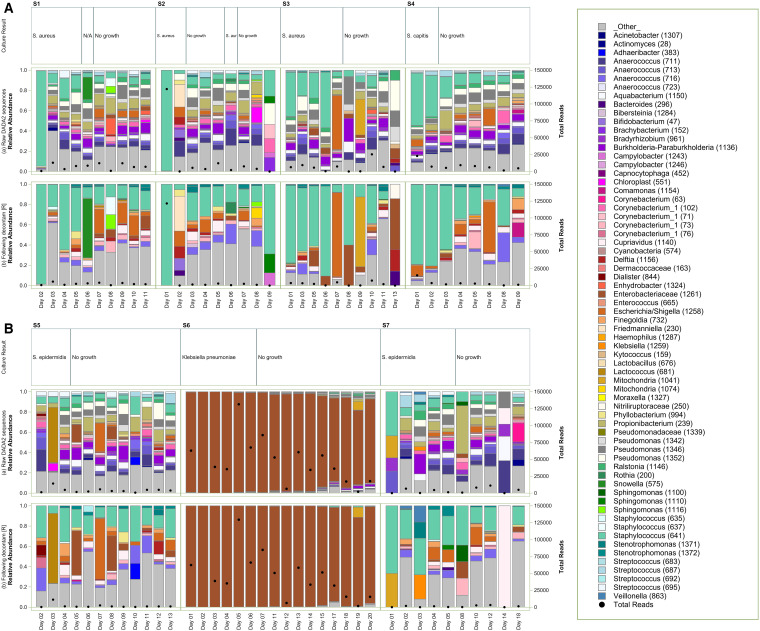
High throughput sequencing results of microbiota for patients with CSF shunt infections. Relative abundance is demonstrated with bars with scale on the left-sided y-axis and total reads are shown with heavy black dots and with the scale on the right-sided y-axis. Bacterial culture results and duration of positive culture results are provided in the upper row. Top panel **(A)** represents raw sequences, and bottom panel **(B)** is following decontam.

Bacterial culture results are compared to HTS and findings from bacterial quantitation by qPCR for the day of infection and the preceding CSF sample in Table 2. In six of the seven CSF shunt infection cases, there was a robust HTS signal (> 10^5^ GE/mL) for the genus of the responsible bacteria in the sample at the time of positive CSF culture. We also observed a decreased proportion of sequences from the genus associated with the responsible bacteria over the course of infection treatment.

## Discussion

In this pilot study, we used rRNA gene sequencing with microbiota analysis and bacterial culture to investigate the progression of CSF ventricular reservoir microbiota and its association with reservoir infection. While a diverse microbial DNA could be detected before ventricular reservoir infection diagnosis, we did not find evidence for the emergence of a dominant organism correlating with that recovered in bacterial culture. We were able to use the same techniques to contrast with the progression of CSF shunt microbiota during CSF shunt infection treatment in infection cases with persistence of bacterial growth from CSF. HTS of 16S rRNA was consistent with culture-based detection methods in CSF infections and correlated with bacterial culture response to antibiotic treatment.

For ventricular reservoir infections, we disproved our hypotheses that (1) increased bioburden and microbial diversity would be associated with longer duration of ventricular reservoir placement and (2) infecting organisms identified by CSF culture would be associated with higher relative abundance of the same genera by HTS. These data suggest limited utility in using these molecular technique(s) for surveillance of ventricular reservoir infection, since ventricular infection appears to emerge abruptly.

For CSF shunt infection, we had hypothesized that (1) infecting organism(s), as identified by traditional culture, would be present at relatively higher relative abundance at the beginning of infection and (2) overall bacterial load and relative abundance of infecting organism would decrease over the treatment course. Using HTS and removing signal from reagents, we continue to demonstrate that numerous bacterial taxa were detected in the CSF of children during CSF shunt infections. The organisms recovered by HTS, including many taxa beyond those recovered by standard aerobic bacterial culture, suggest heterogeneous microbiota. However, the most abundant microbiota identified by HTS results are generally consistent with those identified by culture in CSF shunt infection. The composition of the microbiota changed over the course of infection treatment, likely in response to antibiotic use and surgical removal of the shunt but also potentially because of the introduction of new organisms through external ventricular drain(s) and/or externalized shunt apparatus. Further investigation of the microbiota of the instruments used and/or the skin or body site flora would deepen our understanding of these HTS results. While these results indicate that molecular methods can characterize microbiota dynamics that extend beyond the organisms identified by culture during CSF infections, the clinical relevance of this changing microbiota composition is questionable. There may be a role for molecular diagnostic tools to shorten the duration of intravenous antibiotic treatment for CSF shunt infection cases, but these approaches would require rigorous testing in a clinical trial.

There are several limitations to this pilot work. First, the small sample size makes a formal statistical analysis of our data impractical. Given the variability of microbiome experiments, the strength of any conclusions and the generalizability of our findings are limited. It is, however, valuable to report any findings that could impact patient care while a larger body of clinical data is collected to extend or validate our findings.

The selection of a comprehensive set of appropriate negative controls is difficult. While laboratory-based controls such as kit controls, mock communities, and negative controls were always performed, we recognize challenges associated with low bacterial load in ventricular reservoir and CSF shunt infections. In addition, there are several opportunities for contamination of CSF samples during recovery, storage, and experimentation.^9^ However, sterile conditions were sought throughout recovery and storage of CSF samples, and contamination during experimentation is improbable given little or no detection of DNA in negative controls that were handled identically to and concurrently with the CSF samples. Therefore, contamination from PCR reagents is unlikely to have a major contribution to the bacterial diversity observed. A distinctive “kitome” (sequence reads derived from the DNA extraction kit reagents) was observed. The presence of the kitome may have the potential to affect the interpretation of CSF sample microbiota. In some cases, the sample reads may become indistinguishable from the contaminating kitome reads. Decontam R effectively removed some kitome sequences, and similar results were observed following removal of contaminant sequences detected in extraction kit controls. Differences in HTS recovery have not been noted for samples stored at 4°C.^10^

Another limitation stems from the experimental methods used for microbial identification. Testing from the hospital-certified laboratories was limited to routine aerobic cultures, practices that are subject to individual laboratory protocols, including policies affecting identification of all organisms on a plate, incubation for extended periods of time, and enrichment for slow-growing and fastidious organisms. We did not have information about the variability or density of colonies on the original plates. In addition, anaerobic cultures were not obtained. In addition, the accuracy of species determination could be improved by newer technologies, i.e., MALDI-TOF MS.

While our HTS approach has certain advantages over culture methods in detecting unculturable microorganisms, it has its own limitations. Detection is limited to bacteria, and its resolution is at the genus and sometimes family level. It has a lower sensitivity. False positives could be produced by detection of DNA from nonviable cells, thus not guaranteeing that living bacteria or fungi were present at the time of sample collection. Alternately, HTS may be detecting small colony variants and/or bacteria whose growth is being suppressed by antibiotics.

Despite these limitations, this pilot work contributes to our understanding of the “natural history” of infection in CSF devices. These data suggest limited utility in using these molecular technique(s) for surveillance of ventricular reservoir infection, since ventricular infection appears to emerge abruptly. These data also suggest a return to heterogeneous microbiota in cases of CSF shunt infection when bacterial cultures become negative. We also observe lower microbial loads in CSF than those found in many other infection sample types in these and our earlier studies. Taken together, these pilot results provide supportive rationale for testing the effectiveness of shorter durations of intravenous antibiotic treatment for CSF shunt infection cases in future work that should also address the limitations of the current work and better assess clinical relevance.

## Authors’ Contributions

K.B.W.: Conceptualization, formal analysis, data visualization, writing—original draft. C.E.P.: Formal analysis (lead); writing—review and editing (equal). P.H.: Software (lead); writing—review and editing (equal). D.L.L.: Supervision, writing—review and editing (equal). P.J.McD.: Supervision, writing—review and editing (equal). J.S.H.: Supervision, writing—review and editing (equal). L.R.H.: Formal analysis, resources, supervision, writing—review and editing (equal). T.D.S.: Conceptualization (supporting), funding acquisition, project management, writing—original draft (supporting); writing—review and editing (lead).
